# Indents

**Published:** 1913-10-15

**Authors:** 


### INDENTS.

☞Brief Articles of Technical or Scientific Value will receive notice and comment. Contributions invited.

*A Simple Method of Polishing Instruments.*

Strips of emery and rouge cloth of the finer grades, one and one-half inch wide, folded once lengthwise, and held securely with one end in a small vise or a close fitting work-bench drawer, provide one of the best means for quickly and effectively polishing all kinds of instruments. A stroke similar to that employed in stropping a razor is used, at the same time turning the instrument between the thumb and finger. These strips last a long time, and improve with use.—G. A. Bowman, *Pacific Dental Gazette.*

*Removing Plaster from a Denture.*

The last particles of plaster which may adhere to the unpolished surface of a vulcanite plate can be entirely removed without any difficulty by placing the plate, when polished, into a solution of lysol. The stonger the solution, the less time required for removal of the last trace.—*Edwards Dental Quarterly.*

*Dental Education in United States, as seen by German Educator.*

While medicine has been imported from Europe to America, this the country is the cradle of dental practice and science, and it is interesting to note how the writer though sincerely admiring American dental institutions, believes that in some respects the children have outgrown the foster-mother. In regard to preliminary education, he notes a certain laxity, especially a regrettable ignorance of the classic languages. He believes that the practice of obliging men to attend a prescribed annual course of studies is imcompatible with the idea of academic freedom that governs German universities, and is detrimental to the development of character and self-reliance in the student. The lack of reciprocity in the license to practice dentistry in the various states is criticized as provincial, undignified, and detrimental to a uniformally high professional standard. The great number of demonstrators in the various institutions is recommended on the one hand; on the other, however, the fear is expressed that the instruction suffers in uniformity. As an illustration, the acknowledged inefficiency of orthodontic treatment is cited, the instructor in orthodontia in one of the leading colleges being alleged to have confessed to the writer that hardly two per cent. of the students have any knowledge of orthodontia upon graduation. The lack of knowledge in the field of local anesthesia, of the surgical treatment of pericemental abscess, or surgical prosthesis, and of X-ray work comes in for a large share of censure. The prevalence of the practice of general anesthesia—usually nitrous oxid and oxygen anesthesia—does not meet with the the author's approval, and the lack of interest for surgical prosthesis in cases of fractures and deformities, and for the making of obturators with a view to esthetic and phonetics, appears as startling to the writer as the lack of X-ray apparatus or of efficient appliances for the sterilization of dental instruments in most of the dental schools. These impressions are summarized in the rather left-handed compliment that "If we had come to America twenty-five years ago, before we had our institutions in Germany, the American schools would have made a wonderful impression upon us."

Cohn admires the loyalty of the American patient to his dentist, the practical arrangement of dental offices, and the absence of advertising by ethical practitioners. In regard to the manufacture of dental supplies, he remarks that within the last twenty-five years enormous steps in advance have been made in Germany, so that in a short time the German dentist will no longer be dependent upon the American market. The efforts at public oral hygiene, notwithstanding the Forsyth foundation, seem to be still rudimentary as compared with the institutions established for this purpose in even very small German communities.

All in all, this report is most instructive to the critical and progressive American dentist who is willing to profit from the way in which "others see us," all the more so since the sting of criticism is mitigated by a most charm'ng acknowledgment of the courtesies and hospitality bestowed upon our German colleagues during their sojourn here by federal and municipal authorities, universities, schools, physicians, dentists, and dental dealers and manufacturers.—Dr. K. Cohn, in *Cosmos* for August.

*How Should Root-Canals be Filled.* Mueller makes the following demands upon a good material for root-canal fillings: It must be as nearly permanently antiseptic as possible; it should harden but little, so that it can always be easily removed from the canal if subsequent treatment should become necessary; it should act as a dehydrater, styptic, and mummifier upon the pulp remnants, and should absorb any secretions entering through the apical foramen; it should allow of easy introduction into the narrowest root-canals; it must not produce any discoloration of the tooth; it must not produce mechanical or chemical irritation of the pulp remnants or the pericementum; if forced out through the apical foramen, or into the antrum of Highmore, a fistulous tract, or into an abscess sac, it must not produce any irritation it must not develop any notable amount of vapors or gasses; it must not have a disagreeable odor or taste, it must not decompose in the air; it must be of such consistence that it can be applied without coming in contact with the fingers; it must be applicable in a root-canal that contains a broken broach which canot be removed. All these requirements can be fulfilled only by an antiseptic soft paste which can be introduced under pressure into the root-canals. The paste employed by the author is of the following consistence: Xeroform 5.0., zinc oxid 15.0., glycerin enough to make soft paste; thirty ninims of concentrated carbolic acid. The root-canals are enlarged at their entrance by a suitable bur, and the paste is gently inserted with a syringe, all excess to be removed by cotton pellets, and the root-canal entrance to be closed with a sterilized asbestos disk. If desirable, especially after gangrene of the pulp, a small crystal of thymol may be pressed into the paste at the canal entrance.

The writer employs paraffin to which 30 per cent. iodoform has been admixed only in root-canals of teeth the apex of which is to be resected, also in perforated canals, and as a capping material for exposed pulps that have not been injured so as to produce hemorrhage.—Dr. A. Mueller, in *Cosmos.*

*Teething as a Cause of Disease in Infancy.*

Notwithstanding the remarkable progress that has been made in the science of medicine during the last fifty years, certain traditional beliefs remain as deeply rooted as ever. Not the least noteworthy is the important role which teething is supposed to play in the etiology of the diseases of infancy. As Holt says—"The physician who starts out with the idea that in infants dentition may produce all symptoms, usually gets no further than this in his etiological investigations."

The present views with regard to teething are three: First, there aer those who believe that very many disturbances may be due to teething; second, those who believe that none are due to teething; and third, those who take a middle course, believing that a few disturbances may be caused by teething. To the first group belong a large part of the laity and some physicians. In a recent paper, Neumann has shown in graphic form that in Berlin, from 1877 to 1910, the number of certificates giving teething as a cause of death in infants has diminished from fourteen to three per 1000. The number of certificates giving teething as a cause of death is an index of the intelligence of the physicians, their knowledge of pediatrics being in inverse proportion to the number of such certificates submitted.

The chief danger in believing that many disturbances are due to teething lies in the fact that valuable time may be lost. For example, in cases of acute gastro-enteritis, the disease, falsely attributed to teething, is allowed to progress, and when the child is dangerously ill, possibly beyond all medical help, it is brought for treatment.

The second view is that "teething produces nothing but teeth," that the eruption of the teeth is a physiological process similar to the growth of hair and nails. This view, expressed by Politzer and Fleischmann forty years ago, and more recently by Kassowitz and others, is not incompatible in a somewhat modified form with the third view. So modified, it would read, teething produces nothing but teeth in normal, healthy infants. In such individuals the hair and nails are of normal appearance; but just as in certain constitutional diseases, such as syphilis and cretinism, these parts may show changes, so in infants with certain constitutional peculiarities the eruption of teeth may be sometimes associated with certain disturbances. The fact that an infant has such disturbances may be considered a proof that it is not normal.

It must be remembered that during the period of dentition many other changes are taking place; it is a time of rapid development of nearly all the structures of the organism. In this connection it is noteworthy that the manifestations of the exudative and spasmophilic diathesis, called "Zahnpocken" and "Zahnkrampe" by the Germans, have a tendency to diminish or disappear as the child grows older. "Out growing a disease" is not merely a phrase: in these conditions it actually takes place.

Many of the symptoms attributed to teething are met with in the early months, before the eruption of teeth has begun. Many mothers consider an increased flow of saliva and putting the fingers in the mouth indications of the onset of teething, but countless babies can be observed in which both these symptoms are present quite independently of dentition. The eruption of the permanent teeth is occasionally attended with some disturbance, but how often would one attribute a skin eruption, a diarrhea, or a convulsion in an older child to teething? At a time when teething was considered the cause of a number of infantile disorders, the gums were frequently lanced. Why has this operation been so largely given up? Obviously because it did not meet the indications. It is still done occasionally, and probably often, just as is the cutting of the frenum of the tongue, to ease the mind of the mother.

It is admitted that a few slight disturbances may be caused by teething; that requires no emphasizing. What should be emphasized in the writers opinion is that, in the presence of a disease in an infant, teething should not be diagnosticated as a cause except as a last resort, after every other possible explanation has been excluded.—Dr. Charles Herrman, *Cosmos*.

*A Missing Link.* In the splendid chain of equipment which we have today for saving the natural teeth when they are attacked by caries, there is still one deficiency which must yet be made good. We have at our command gold and porcelain inlays, foil fillings, amalgam, gutta percha and the cements, each of which in its way is worthy of due consideration. In fact it may be said that if teeth are taken in time they need never be lost through dental caries.

But there is one situation which to meet perfectly taxes all of our ingenuity, and for which we yet lack the proper equipment. This relates to cavities in the anterior teeth which are exposed to view. It is not enough that we are able to save these teeth—we can do this with gold foil or in some instances with gold or porcelain inlays, but in our modern civilization the necessities of the case demand that we shall save them without displaying any evidence of our operations. This we cannot do with any of the materials at our command. It is true that porcelain has long been heralded as the *ne plus ultra* of perfection so far as esthetics was concerned, but even porcelain cannot be held without fault in this particular. In many cases we know that when porcelain is first inserted the esthetic effect is good but in a few years in most mouths the tell-tale line of junction between porcelain and enamel shows up to mock our efforts. It is safe to say that less porcelain is being used today than five years ago, and it is largely due to the disappointment in results after years of service.

The thing at present which seems to be largely supplanting porcelain for filling anterior teeth is the silicate cement in one form or another. This material may be said to be passing through its period of test, and no man can predict with accuracy what its future may be. It assuredly can be made more satisfactory in appearance than even porcelain, and the great question which looms high on the horizon of every conscientious practitioner is as to its ultimate permanence. In some instances it promises sufficiently in this respect to justify us in using it, but always with the mental reservation that we dare not promise the patient how long it will remain satisfactory. To be always puttering with a material of which we are never certain is a sad blow to our highest ideals, and the fact remains that today we are suffering from a missing link in the armamentarium with which we are equipped to save teeth.

Who will be the genius to supply dentistry with this great need?—Editorial in September, *Dental Review*.

*To Cleanse Vulcanite Dentures.* Add a little soda bicarbonate to a tumbler nearly full of rain water then add a few drops of sulfuric acid and place the plate in the nickel vessel quickly. The effervescence will quickly destroy the glutinous mouth deposits of all kinds. —*The Dental Brief Sept. 1914.*

